# Additive Effect of Botanical Insecticide and Entomopathogenic Fungi on Pest Mortality and the Behavioral Response of Its Natural Enemy

**DOI:** 10.3390/plants9020173

**Published:** 2020-02-01

**Authors:** G. Mandela Fernández-Grandon, Steven J. Harte, Jaspher Ewany, Daniel Bray, Philip C. Stevenson

**Affiliations:** 1 Natural Resources Institute, University of Greenwich, Central Avenue, Chatham Maritime, Kent ME4 4TB, UK; 2 Royal Botanic Gardens, Kew, Richmond, Surrey TW9 3DS, UK

**Keywords:** biopesticide, organic pesticide, Y-tube olfactometer, pyrethrum, parasitoid, entomopathogenic fungi, leaf disc assay, insect behavior, survival analysis

## Abstract

Sustainable agricultural intensification employs alternatives to synthetic insecticides for pest management, but these are not always a direct replacement. Botanical insecticides, for example, have rapid knockdown but are highly labile and while biological pesticides are more persistent, they are slow acting. To mitigate these shortcomings, we combined the entomopathogenic fungus (EPF) *Metarhizium anisopliae* with pyrethrum and evaluated their efficacy against the bean aphid, *Aphis fabae*. To ascertain higher trophic effects, we presented these treatments to the parasitoid, *Aphidius colemani,* on an aphid infested plant in a Y-tube olfactometer and measured their preferences. Aphid mortality was significantly higher than controls when exposed to EPF or pyrethrum but was greater still when exposed to a combination of both treatments, indicating an additive effect. This highlights the potential for applications of pyrethrum at lower doses, or the use of less refined products with lower production costs to achieve control. While parasitoids were deterred by aphid infested plants treated with EPF, no preference was observed with the combination pesticide, which provides insight into the importance that both application technique and timing may play in the success of this new technology. These results indicate the potential for biorational pesticides that combine botanicals with EPF.

## 1. Introduction

Pesticidal plant extracts are an important component of sustainable integrated pest management (IPM) and can offer an effective alternative to synthetic pesticides for management of pests, especially for smallholders [1]. Pesticidal plants typically have lower impact on higher trophic levels, including natural enemies of pests, so are better suited to sustainable production [2,3] and are locally available at a lower cost than synthetic chemicals [4]. The most important commercial botanical pesticides include pyrethrum and neem products [5]. Pyrethrum is a natural insecticide extracted from the flowers of *Chrysanthemum cinerariaefolium* and *Chrsanthemum cineum* [6,7] which has been used for controlling field, household, and storage pests, and parasites in livestock and humans [8,9,10]. A combination of awareness of the negative impacts of synthetic pesticides and increased pressure from regulatory authorities on permitted chemicals has seen the global demand for biopesticides grow over the past decade at an estimated 15% per annum [11,12,13], compared to 3% per annum for synthetic pesticides [11].

Natural pyrethrum contains six entomotoxic compounds: cinerin I and II, pyrethrin I and II and jasmolin I and II [14]. Pyrethrins enter the insect body via ingestion or contact, penetrate the epidermis and are distributed throughout the body in the haemolymph [7]. The insecticide disrupts the insect’s peripheral and central nervous systems by causing repetitive discharges of nerves, resulting in paralysis [15]. Pyrethrins have a rapid “knockdown” effect preceding insect death [7,14] and insects usually die in a few minutes or hours following exposure to a fatal dose [8].

Pyrethrins influenced the development of some of the most widely used synthetic insecticides—the pyrethroids, including cypermethrin, permethrin, deltamethrin, fenvalerate and bifenthrin. The drive towards different synthetic pyrethroids was to increase their stability in the environment, providing effective control for longer. This has proven successful as pyrethroids are routinely used as agricultural insecticides with high adoption rates internationally [8].

The active components in pyrethrum are highly labile in ultraviolet (UV) light, non-persistent, and are less toxic to humans and the environment [9,16,17,18]. Although the lack of persistence has previously limited use of natural pyrethrum as an agricultural insecticide [8,15] it is less disruptive to IPM programs that include beneficial insects than conventional insecticides.

One of the emerging technologies as part of IPM is the use of entomopathogenic fungi (EPF) [19], which can be used to control a wide range of agricultural pests [20]. They have no negative effects on human health [21]. Entomopathogenic fungi are specific pathogens of insects that can infect their hosts through the external cuticle [20]. Rapid penetration and infection of a susceptible host occurs at high humidity [20], but spores can remain viable on the cuticle during unfavorable conditions and penetrate when humidity rises, even if only for a short time [22].

Upon successful penetration, the fungi develop, colonize the insect’s internal organs and the insect eventually dies. It is after insect death that hyphae emerge, followed by spore formation and production of numerous conidia on the cadaver [20]. EPF, such as *Metarhizium anisopliae* (Metschnikoff) used in this study, can only complete their lifecycles and increase populations by producing numerous conidia after the death of infected hosts [23] which will disperse, infecting more insects and continue the propagation cycle. 

There are successful synergies and compatibilities of EPF with different plant-based pesticides for improved pest control. For instance, the combination of sub-lethal doses of the EPF *Beauvaria bassiana* and neem extract increased mortality against whitefly, *Bemisia tabaci,* nymphs when neem insecticide was drenched with simultaneous application of *B. bassiana* on tomatoes plants [24,25]. *Beauvaria bassiana* (isolate PL63) was compatible with botanical extracts from *Trichilia catigua* leaves and effectively controlled insect pests in Brazil [26]. Another study by Shoukat et al. [27], revealed that both *M. anisopliae* and *B. bassiana* showed a synergistic effect when mixed with neem extract, *Azadirachta indica,* and increased mortality against 3rd instars of *Culex pipiens* in the field. These examples of the compatibility of EPF with pesticidal plant extracts suggests potential in combining EPF and pyrethrum to improve efficacy through the rapid knockdown of pyrethrum and the persistent control offered by EPF to control agricultural pests and combat pest resistance.

A benefit that may be realized through a combination biopesticide over synthetic pesticides could be reduced impact on beneficial insects in the environment through decreased exposure and greater specificity. EPF has been reported to be pathogenic to beneficial insects, including parasitoids. *B. bassiana* was found to infect and kill adult *Aphidius colemani* with infection rates ranging from 46.3% to 60% [28] and between 57.6 to 66% [29] under greenhouse environments. Shipp et al. [29] did not recommend the use of adult *A. colemani* together with *B. bassiana* for pest control in greenhouses. The exposure of *A. colemani* to different EPF strains such as *M. anisopliae* is still to be explored.

However, time of application of EPF has been manipulated to reduce parasitoid mortality and affect the use of EPF together with parasitoids in pest control. For instance, parasitoid *Aphidius matricariae* and *B. bassiana* (strain EUT116) were effective against the aphid, *Myzus persicae* when the fungus was applied 96 hours after the release of parasitoids [30]. *Beauvaria bassiana* (strain PL63) and the aphid parasitoid, *Diaeretiella rapae* were recommended against *M. persicae* [31]. Another study by Mohammed and Hatcher [32], revealed that introducing EPF *Lecanicillium muscarium* six days after releasing *A. colemani* was effective against *M. persicae* in a greenhouse environment. Based on these results, usage of selected EPF isolates and applying EPF after parasitism can reduce detrimental effects on parasitoids. However, to inform the effective use of EPF with the beneficial insects, it is important that we understand their interaction with the fungi.

In this study, we assessed the efficacy of pyrethrum, *Metarhizium anisopliae* EPF and the combination of both on mortality of the aphid pest, *Aphis fabae*, and how the aphid parasitoid, *Aphidius colemani,* responded to the odors associated with these compounds. The components were selected for this proof of principle because of the ready availability of pyrethrum in low-income areas and the known efficacy of this *M. anisopliae* strain against this highly problematic aphid pest. To gauge the potential in establishment of EPF on the aphid population, we also recorded incidence of visible fungal establishment with hyphae seen emerging from the cuticle and the number of offspring produced by the aphids following exposure. We found that both pyrethrum and the EPF led to a significant increase of mortality on *A. fabae* which was further enhanced when they were presented in combination. Visible fungal growth occurred more rapidly on aphids treated with the combination biopesticide, indicating establishment in population could be accelerated through the multimodal action. It was also shown that its associated parasitoid, *A. colemani*, preferentially selects plants that do not contain EPF when foraging using odor, however, the preference exhibited is absent in the combined treatment.

## 2. Results

### 2.1. Aphid Mortality Assay

#### 2.1.1. Survival

**Figure 1:**
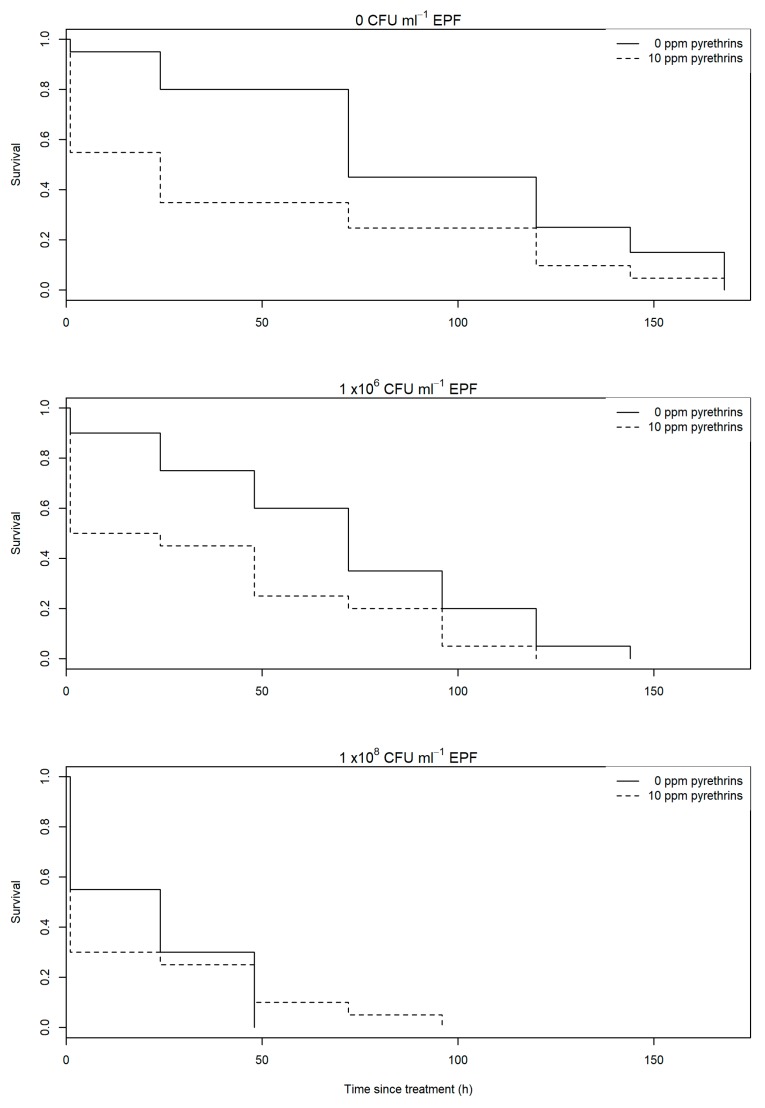
Survival of individual *A. fabae* exposed to *M. anisopliae* alone (solid line) or in combination with pyrethrum (dotted line). Entomopathogenic fungus (EPF) was tested at 0 colony forming units (CFU) mL^−1^ (carrier oil only, top graph), 1 × 10^6^ CFU mL^−1^ (middle graph) and 1 × 10^8^ CFU mL^−1^.

No significant interaction was found between the application rate of EPF 0 CFU mL^−1^ (carrier oil only), 1 × 10^6^ CFU mL^−1^ or 1 × 10^8^ CFU mL^−1^ and pyrethrum presence (10 ppm pyrethrins) or absence (0 ppm) on aphid survival (χ^2^ = 0.70, df = 2, *p* = 0.70). However, significant independent effects of both pyrethrum (χ^2^ = 6.56, df = 1, *p* = 0.01) and EPF concentration (χ^2^ = 16.8, df = 2, *p* = 0.001) were found on aphid survival (Figure 1). Addition of pyrethrum led to a 40.5 h reduction in predicted mean aphid survival at 0 CFU mL^−1^ EPF (from 80.1 h to 39.6 h). At 1 × 10^6^ CFU mL^−1^ survival was reduced by 29.2 h through addition of pyrethrum (from 67.3 h to 35.3 h), and by 10 h at 1 × 10^8^ CFU mL^−1^ (from 19.7 h to 9.7 h). Survival was reduced significantly through addition of EPF compared to No EPF (χ^2^ = 6.9, df = 1, *p*=0.009), and from 1 × 10^6^ CFU mL^−1^ to 1 × 10^8^ CFU mL^−1^ EPF (χ^2^ = 9.9, df = 1, *p* = 0.002).

#### 2.1.2. Hyphal Growth on Insect Surface

**Figure 2:**
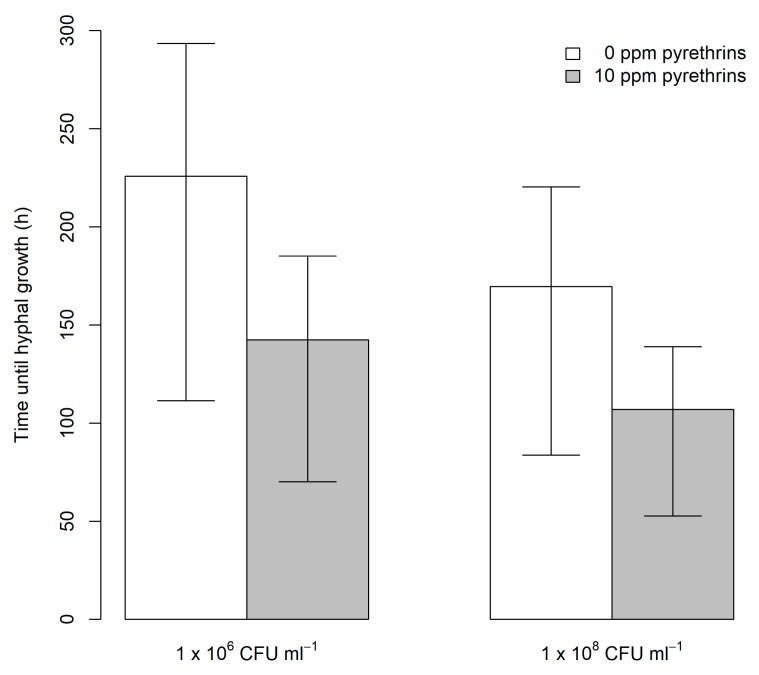
Predicted time (± upper and lower quantiles) until hyphal growth was observed on individual *A. fabae* exposed to *M. anisopliae* alone or in combination with pyrethrum. CFU: colony forming units.

Overall, aphids (*n* = 40) which had not been treated with EPF (0 CFU mL^−1^) showed no hyphal growth up to 192 h after treatment. Aphids not treated with EPF were therefore excluded from further analysis of time until visible fungal growth. Increasing concentration of treatment from 1 × 10^6^ CFU mL^−1^ to 1 × 10^8^ CFU mL^−1^ significantly reduced time until hyphal growth (χ^2^ = 10.74, df = 1, *p* = 0.001). Addition of pyrethrum at 10 ppm pyrethrins also significantly reduced time until hyphae formation was observed (χ^2^ = 10.74, df = 1, *p* < 0.001). However, no interaction was found between EPF level and pyrethrum treatment on time until hyphal growth was observed (χ^2^ = 2.37, df = 1, *p* = 0.12). Addition of pyrethrum reduced predicted mean time until growth was seen by 84 h at 1 × 10^6^ CFU mL^−1^ (from 226 h to 142 h) and 63 h at 1 × 10^8^ CFU mL^−1^ (from 170 h to 107 h) (Figure 2).

#### 2.1.3. Number of Offspring

**Figure 3:**
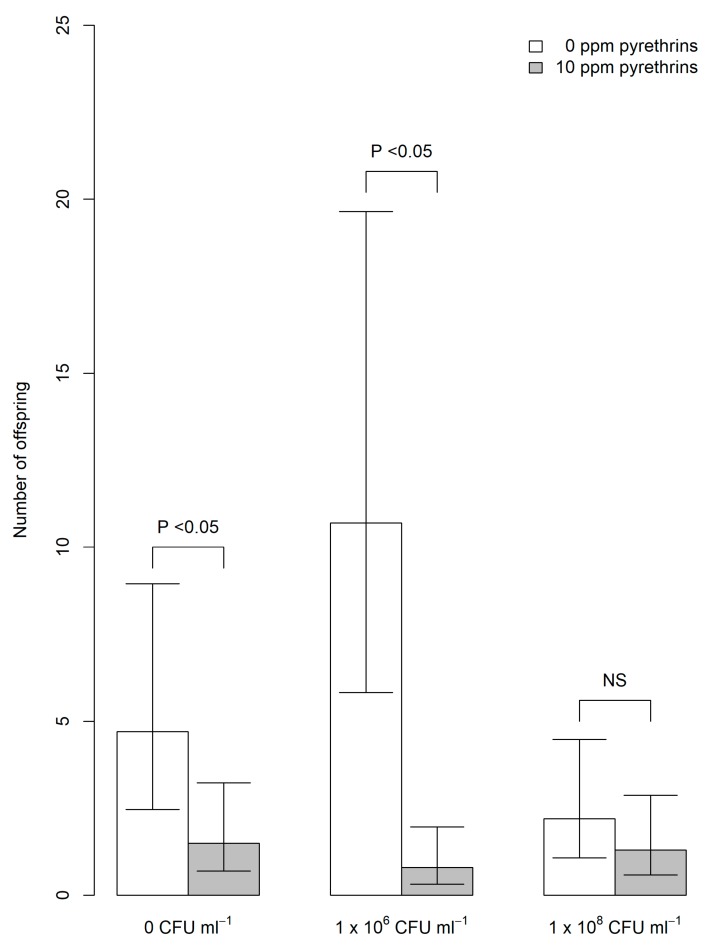
Predicted mean number (± 95% confidence intervals) of offspring produced by individual *A. fabae* exposed to *M. anisopliae* alone (white bars) or in combination with pyrethrum (10 ppm pyrethrins; grey bars). EPF was tested at 0 colony forming units (CFU) mL^−1^ (carrier oil only, left), 1 × 10^6^ CFU mL^−1^ (middle) and 1 × 10^8^ CFU mL^−1^ (right).

Due to a very low offspring count in the first block of replicates, only aphids tested in the second block of treatments were used in the analysis. In this block, a significant interaction was found between EPF level (0 CFU mL^−1^ (carrier oil only), 1 × 10^6^ CFU mL^−1^ or 1 × 10^8^ CFU mL^−1^) and pyrethrum presence (10 ppm pyrethrins) or absence (0 ppm) on total number of offspring produced by each aphid (χ^2^ = 7.01, df = 2, *p* = 0.03). At 0 CFU mL^−1^ and 1 × 10^6^ CFU mL^−1^, addition of pyrethrum resulted in significantly fewer offspring produced (Tukey’s test, *p* < 0.05, Figure 3). However, no significant effect of pyrethrum was found on number of offspring produced by aphids exposed to 1 × 10^8^ CFU mL^−1^ of EPF. 

### 2.2. Parasitoid Dual-Choice Assays

**Figure 4:**
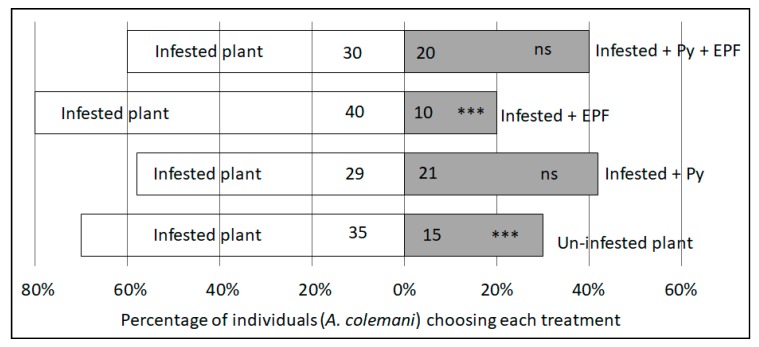
Parasitoid, *A. colemani*, responses to treatments in a Y-tube olfactometer. N = 50 for each treatment, with the number of individuals choosing each side indicated on the relevant bar. Where ‘Infested’ refers to aphid colonized plant, Py is treatment with pyrethrum and EPF is treatment with entomopathogenic fungi, *Metarhizium anisopliae* (ICIPE 62) *** Indicates *p* < 0.01, ns = not significant.

When presented with the choice of aphid-infested or uninfested plant material through a dual-choice assay, more female parasitoids chose the aphid-infested material (exact binomial test, *n* = 50, *p* = 0.0066) (Figure 4). This served as a positive control to confirm that parasitoids would orientate towards *A. fabae* in the absence of visual cues. All other experimental treatments used only aphid-infested bean plants. When presented with a choice between two aphid-infected plants, one of which had been treated with pyrethrum, no significant difference was found between proportion of parasitoids choosing an untreated plant, and a plant which had been treated with pyrethrum (exact binomial test, *n* = 50, *p* = 0.322). However, significantly fewer parasitoids chose the EPF-treated compared to the untreated plant (exact binomial test, *n* = 50, *p* < 0.001). When the pyrethrum was presented alongside the EPF and compared to an untreated plant, the parasitoid displayed no preference (exact binomial test, *n* = 50, *p* = 0.203) (Figure 4). For all the replicates there was only one non-responder recorded for failing to make a choice in the allotted time (treatment containing pyrethrum + EPF), this individual was excluded from the analysis.

## 3. Discussion

Our working hypotheses for this study were:

1. Efficacy of pyrethrum and EPF would be enhanced when presented in combination.
2. The biopesticides would affect parasitoid plant/host preference.

Through the evaluation of aphid mortality, hyphae formation and offspring production after exposure to the biopesticides, we found that efficacy was enhanced when the components were presented in combination. This additive effect of combination was observed through reduced survival, more rapid formation of hyphae and reduced fecundity. Increased mortality was recorded as EPF concentration increased, though from a practical perspective, the level of mortality achieved with lower dosage may be sufficient in pest control programs and may even be preferable if it permits low-level persistence of the host for biocontrol agents. One of the difficulties in controlling aphid populations lies in their high rate of fecundity. The significant reduction in fecundity noted with exposure to the combination treatment could be critical in more effective control as it may curb the exponential rate of population growth. However, in this study only ten replicates were evaluated for offspring production in each treatment. This was due to only four offspring being produced in the initial block of replicates. It is not clear why this number was this low. To evaluate this further we suggest the experiment be repeated and assessment to include the intrinsic rate of increase and other population metrics to gain greater insight into how this is likely to affect population dynamics.

The increase in mortality may be due to the bimodal effects of the combined treatment as the immediate attack on the nervous system provided by the pyrethrum leave individuals more susceptible to infection from the EPF. This concurs with previous studies on pests that have shown additive or synergistic effects when EPF and pesticidal plant products are presented in combination [33,34,35]. In addition to the increased mortality with a combined biopesticide application, it was notable that the time lag before hyphae were observed was shortened in the lower dosage application (226 h to 142 h). This difference of 84 h could have a considerable impact on the viability of such a product and especially when considered over multiple generations of the pest. One of the most widely recognized drawbacks to EPF application is the slow-acting nature of the product [35,36,37,38]. Although it can offer sustained control, this relies on its establishment within the population through propagation via the host. A guiding principle behind the exploration of a combined biopesticide is for a product that overcomes the short-lived nature of pyrethrum and the slow-acting nature of the EPF. An accelerated rate of establishment, as indicated by this study, could be a critical advantage to such a product. However, we recognize that this is a laboratory evaluation of the interaction under controlled conditions and with limited replication. The real impact of this would need to be established through longer running trials which assess the formation of conidia from the host and population suppression over multiple generations in a more field-realistic situation.

The results of the mortality experiments show strong support for the development of combined biopesticides as a new tool for IPM. In addition to a direct increase in pest mortality, there are indications that propagation of the EPF may be occurring more rapidly and fecundity of the pest is being suppressed. Furthermore, it is important to note the pyrethrin dosages applied in the trials (10 ppm) were a fifth of that recommended for effective pest control in the field. This was used to allow any potential synergy or additive effect of combination to be recorded as it was identified in preliminary trials that mortality was too high at 50 ppm to determine these effects. The mortality observed at this low dosage of pyrethrins when presented in combination with the EPF has greater practical and commercial appeal for this technology. Refinement of pyrethrum remains a relatively expensive process and one limited by the technology available in an area. The use of less refined material could lead to lower cost production, a reduction of impact on non-target species and greater potential for formulation in and for lower income countries. We also identify that there are various shortcomings to the use of these biopesticides which a combination approach will need to overcome and will assess through field trials.

Evidence from the Y-tube olfactometer behavioral assays supported our second hypothesis that the behavior of the parasitoid would be affected by volatiles from treated materials. The control demonstrated that the parasitoid was able to detect *A. fabae* feeding on the bean plants and would move towards them, behaviors indicative of foraging. When EPF was applied as the only variable present, wasps showed a preference for the plant-aphid treatment without the EPF. The avoidance of EPF by natural enemies has previously been observed in studies looking at ladybirds, *Coccinella septempunctata,* and anthocorids, *Anthocoris nemorum* [39,40] respectively. The behavior possibly confers fitness benefits as the wasp may reduce its exposure to the fungal pathogen. However, studies on parasitoid *Cephalonomia tarsalis* showed no avoidance behavior in response to the EPF *B. bassiana* [41]. The avoidance behavior of parasitoids to EPF may be species dependent, which highlights the importance of study on commercially relevant organisms. It is interesting to note that EPF avoidance observed in this study was absent in the presence of pyrethrum which could indicate either an interaction between the components or the perception of the EPF-aphid-plant treatment.

Future research should consider other aspects of the behavior of beneficial insects and elucidate the mechanisms behind this observed preference for non-EPF treated material. Experiments could be performed to disentangle the interaction between plants, aphids and EPF to identify whether individual components or the interaction is responsible for the deterrence that was observed. Next steps in this direction would be to evaluate the direct impact of relevant EPF strains to the parasitoids, the odors involved in potential repellency from EPF and how these behaviors affect success in field settings.

Our findings align with what has been found previously that plant-based insecticides can be complemented by addition of EPF. The full potential of such a technology is still to be explored and different formulations should be investigated using different EPF and botanical components. It is also important that these experiments are taken out of the laboratory and into the field to assess their efficacy in highly variable field conditions. In future testing we also suggest that the impact on beneficial insects in the environment is considered as a high priority and should extend to include pollinators as well as natural enemies. Their susceptibility to the combined formulations should be assessed and the findings should inform the future use of this technology.

## 4. Materials and Methods 

### 4.1. Insect Rearing

Aphids, *Aphis fabae,* were obtained from colonies at Harper Adams University. The population was maintained on potted broad bean plants at temperatures of 27 °C, on a 12 h, L:D cycle. Fresh plant material was introduced into the colony if alatae were seen to form with old material removed following 24 h.

Parasitoid species, *Aphidius colemani,* were used in laboratory bioassays. The parasitoids were obtained from Bioline Agro-Sciences Ltd, UK from a different rearing background to that used in experiments. It has previously been found that parasitoids 2 to 5 days old display greater fecundity and higher rates of parasitism [42,43,44]. For these experiments female *A. colemani* 3 to 5 days old were used to increase the likelihood of host seeking behavior.

### 4.2. Entomopathogenic Fungi

The commercially available strain of *Metarhizium anisopliae* isolate, ICIPE 62, in an oil emulsion was obtained from Real IPM Kenya (Madaraka, Thika, Kenya) and used for the experimental work. Dilutions were conducted as necessary to obtain the concentration of colony forming units (CFU) required for trials.

### 4.3. Preperation of Pyrethrum

Semi-refined pyrethrum product was supplied by Botanical Extracts EPZ Ltd (Twiga Crescent, Export Processing Zone, Athi River, Kenya) for use in the experimental work. The product was analyzed to ensure the correct dosage of active ingredient was used in experimental work. Pyrethrins were analyzed using an Agilent 1200 series HPLC system (Agilent Technologies, Santa Clara, United States) consisting of modular quaternary pump, degasser, auto-sampler, column oven and photodiode array detector. Separation of entomotoxic pyrethrum constituents was achieved on a Waters X-Select T3 column (250 × 4.6 mm, 3.5 μm) and a guard column with the same characteristics, all kept at 25 °C. The chromatographic conditions were: flow rate 1 mL min^−1^, sample injection volume of 10 μL and mobile phases; A (100% H_2_O), B (100% MeOH) and mobile phase C (1% formic acid). 27/68/5 (A/B/C) which was held for 2 mins, raised to 5/90/5 over 22 min (24 min total), followed by wash and re-equilibration steps. The photodiode array detection was conducted by scanning between 200 and 600 nm. 

Individual compounds in each sample were identified by comparing their retention times and UV–Vis spectra with those of a standard pyrethrum sample purchased from Sigma (Gillingham, Dorset, UK). Quantitative determination of the target compounds in the extracts was performed using external calibration curves in the concentration range of 0.05 to 1 mg mL^−1^ (detection at 225 nm).

Total w/w% of the active compounds was 17%, comprised of cinerin I (0.5%), cinerin II (3.7%), pyrethrin I (0.5%), pyrethrin II (1.4%), jasmolin I (11%) and jasmolin II (1.5%). Retention times were 18.9, 11.4, 19.1, 11.8, 22.3 and 14.1 minutes respectively. 

The pyrethrum extract was subsequently diluted with deionized H_2_O, without the need for surfactants or adjuvants, to the required ppm values of pyrethrins, as specified in the mortality assay methods. All ppm values quoted in this section are for total pyrethrin content i.e., cinerin I & II, pyrethrin I & II and jasmolin I & II.

### 4.4. Mortality Assays

Leaf discs were removed from broad bean leaves using a sterilized 26 mm cork bore. Discs were then dipped into either negative control (H_2_O), low concentration of EPF (1 × 10^6^ CFU mL^−1^), high concentration of EPF (1 × 10^8^ CFU mL^−1^), pyrethrum (10 ppm pyrethrins), low dose combination (EPF at 1 × 10^6^ CFU mL^−1^ with 10 ppm pyrethrins) or full combination dose (EPF at 1 × 10^8^ CFU mL^−1^ with 10 ppm pyrethrins). The concentration applied of pyrethrins is lower than the recommended 50 ppm for aphid control. A lower concentration of 10 ppm was selected from preliminary trials demonstrating that this elevated mortality but did not lead to 100% mortality, making it a viable candidate to assess interaction with the fungi.

During the preparation of leaf discs, a 1.5% agar solution (Oxoid Technical Agar No. 2) was prepared using distilled water. Once the solution was fully mixed, 10 ml was decanted into 29 mL pots (4.5 cm height × 4 cm diameter) to cool. Once the solution was viscous but not completely set, leaf discs were embedded into the agar ensuring the edges were sufficiently covered. 

A single adult *A. fabae* was gently removed from plants using a fine paintbrush and placed onto the center of each leaf disc. A partially mesh lid was placed on each pot preventing aphid escape while avoiding a build-up of moisture. Pots were placed onto the trays in a Latin square design which was altered with each replicate to reduce positional bias. Treatments were maintained in a 26 °C room, on an 12:12 L:D light cycle.

All samples were monitored daily with counts made for offspring which were removed as they were found, mortality of the adults, and visible formation of hyphae. Monitoring was conducted at the same time on each day with the same experimenter conducting the observations to ensure consistency. A total of 20 replicates were performed for each treatment. The experiment was conducted across two blocks.

### 4.5. Parasitoid Choice Assays

Behavioral response of *A. colemani* to treatments of aphid-infested plants (positive control), uninfested plant (negative control), aphid-infested plant + pyrethrum, aphid-infested plant + EPF, and aphid-infested plant + pyrethrum + EPF was tested using a dual-choice assay.

Three days prior to each experimental day, bean plants were transferred from glasshouse to the bioassay room. Broad bean, *Vicia faba*, Dwarf Sutton variety (Kings seeds, Colchester, UK) were used for all experiments. All plants were used 3 weeks after germination with between 4 to 5 leaf pairs developed. Using a fine paintbrush, 50 aphids of mixed ages where transferred from the colony onto fresh bean plants and left for 72 h to establish plants in the ‘Infested’ treatments. This level is sufficient to induce production of defensive plant volatiles [45]. Uninfested control plants were maintained in the same condition with no direct contact with aphids. The pyrethrum and EPF suspension were placed in separate 1 L spray bottles and sprayed on the plants in a closed arena. All plant leaves were sprayed both on the top and the underside with pyrethrum or EPF to reflect application of these materials in a field environment. After administering the treatment, plants were left for 15 min before conducting the experiments.

In the treatment combining EPF and pyrethrum insecticide, equal amounts of each were sprayed separately. The order of spraying was alternated between trials. The sprayed plants were then placed in separate glass vessels to conduct the experiment. All the treatments were tested against the positive control to make four treatment sets per day.

A glass Y-tube olfactometer (stem 8 cm, arms 8 cm, internal diameter 1 cm and 120° angle between arms) was used to assay parasitoid response to plant treatments in the absence of visual stimuli. The Y-tube was connected to two, 3 L Kilner jars containing the positive control and the other contained either an experimental treatment or negative control. The Kilner jars used were modified with an airtight inlet and outlet fittings, allowing a continuous flow of charcoal filtered air at 200 mL/min through each vessel to the two arms of the Y-tube olfactometer via Teflon tubing.

Experiments were conducted during active foraging times for the parasitoid (4–9 h after scotophase). During the experiment, individual *A. colemani* were released using a 1 ml pipette head connected to the base of the Y-tube olfactometer and removed immediately after the parasitoid entered the tube. Each *A. colemani* was observed for a maximum of 5 min or until it travelled 6 cm up one of the Y-tube arms and remained there for 30 s. Wasps that did not enter the Y-tube after 5 min were recorded as non-responders.

All the four treatments were tested on each experimental day, with the order determined using a Latin square. The bioassay arena of the Y-tube blocked the entrance of light from all sides except from the direction of the Y-tube olfactometer arms. In each treatment, the Y-tube arms and odor sources were swapped after five parasitoids were tested to minimize directional bias or any bias of choice due to light in one arm. A different Y-tube was used in each experiment, and all Y-tubes were cleaned with 70% ethanol and left to dry before use. Each parasitoid was used only once, reflecting a true biological replicate. Fifty replicates were completed for each treatment.

### 4.6. Statistical Analyses

#### 4.6.1. Survival

The first objective of statistical analysis was to determine whether application of pyrethrum and EPF would result in lower aphid survival than treatment with EPF alone. Differences between treatment groups were first visualized by plotting Kaplan–Meier survival estimates (Figure 1). A model with Weibull errors was used to test whether there was an interaction between EPF level (0 CFU mL^–1^, 1 × 10^6^ CFU mL^−1^ or 1 × 10^8^ CFU mL^−1^) and presence (10 ppm pyrethrins) or absence (0 ppm) of pyrethrum on aphid survival. The time interval in which the aphid died was entered as the dependent variable. Aphids which did not die during the experiment were recorded as censored cases. Independent effects of EPF level and pyrethrum presence or absence were then tested separately. Differences in survival between individual EPF levels were tested through model simplification (presence vs. absence of EPF, low (1 × 10^6^ CFU mL^−1^) vs high (1 × 10^8^ CFU mL^−1^) EPF. Significance of all effects was assessed through χ^2^ test changes in residual deviance following deletion from the model [46]. All analyses were performed in R [47,48,49]. 

#### 4.6.2. Visible Fungal Growth

A model similar to the survival analysis was applied with Weibull errors was used to determine whether application of pyrethrum would result in faster hyphae formation of EPF at the three levels of EPF tested. In this model, time interval in which hyphae were first observed was entered as the dependent variable. Replicates in which spores were not recorded were entered as censored cases. EPF level and pyrethrum treatment were entered as factors in the model. An interaction was included to determine whether the effect of pyrethrum on time until hyphae emergence varied with initial EPF concentration. 

#### 4.6.3. Number of Offspring

A generalized linear model with negative binomial areas [50] was used to determine whether application of pyrethrum and EPF had significant effects on aphid offspring production. Total number of offspring produced by each aphid across the experiment was entered as the dependent variable. EPF level (0 CFU mL^−1^, 1 × 10^6^ CFU mL^−1^ or 1 × 10^8^ CFU mL^−1^) and pyrethrum level (0 ppm pyrethrins or 10 ppm pyrethrins) were entered as factors in the model. An interaction term was included to test whether the effect of pyrethrum on number of offspring produced varied with EPF level applied. Significance of each term was assessed through likelihood ratio tests (χ^2^) following deletion from the model. Differences between individual factor levels in numbers of offspring produced were assessed through Tukey’s tests performed on estimated means [51]. Analysis was restricted to the second round of aphids tested, as none of the aphids in the first round produced offspring.

#### 4.6.4. Parasitoid Dual-Choice Assays

Choice assays using the Y-tube olfactometer data was analyzed using two-sided exact binomial tests, with an assumption of a 50:50 distribution if the wasps were moving at random. Wasps which made no choice after 5 minutes were excluded from analysis. Tests were conducted using R (v 3.5.1, Vienna, Austria).

## 5. Conclusions

Here we show that a combination of the entomopathogenic fungi, *Metarhizium anisoplae,* and pyrethrum led to a higher rate of mortality in *Aphis fabae* than for the individual pesticides when tested alone. Thus, the combination of these two biopesticides was effective at killing the target pest more effectively than the individual components with no apparent contraindications, illustrating a novel approach to compensate for their individual shortcomings; the rapid breakdown of pyrethrum and slow activation of EPF. The development of fungi on the external cuticle was also observed earlier when EPF was presented with pyrethrum, which may be key to more rapid establishment in the population. A surprising finding was that when used alone, the EPF was repellent to the parasitoid *Aphidius colemani,* however, when presented in combination with the pyrethrum had no effect on foraging behavior of the natural enemy. Thus, the combination of EPF and pyrethrum may be better suited to use in an IPM system that included natural enemies, though timing considerations may be critical. Combinations of biopesticides that have different mechanisms of action have the potential to improve the efficacy of the individual components and reduce the build-up of resistance. The additive effect also suggests that there is potential for applications of pyrethrum at lower doses and so reducing effects at higher trophic levels or that less refined products could be used at lower production costs to achieve control. Studies of behavior provide insight into the importance that application technique and timing play in the effectiveness of this novel combination pesticide technology.
